# 320-nm Flexible Solution-Processed 2,7-dioctyl[1] benzothieno[3,2-b]benzothiophene Transistors

**DOI:** 10.3390/ma10080918

**Published:** 2017-08-09

**Authors:** Hang Ren, Qingxin Tang, Yanhong Tong, Yichun Liu

**Affiliations:** Centre for Advanced Optoelectronic Functional Materials Research and Key Laboratory of UV-Emitting Materials and Technology, Northeast Normal University, Ministry of Education, Changchun 130024, China; renh221@nenu.edu.cn (H.R.); tongyh@nenu.edu.cn (Y.T.); ycliu@nenu.edu.cn (Y.L.)

**Keywords:** organic thin-film transistors (OTFTs), ultrathin, flexible, conformal, solution-processed, 2,7-dioctyl[1]benzothieno[3,2-b]benzothiophene (C8-BTBT)

## Abstract

Flexible organic thin-film transistors (OTFTs) have received extensive attention due to their outstanding advantages such as light weight, low cost, flexibility, large-area fabrication, and compatibility with solution-processed techniques. However, compared with a rigid substrate, it still remains a challenge to obtain good device performance by directly depositing solution-processed organic semiconductors onto an ultrathin plastic substrate. In this work, ultrathin flexible OTFTs are successfully fabricated based on spin-coated 2,7-dioctyl[1]benzothieno[3,2-b]benzothiophene (C8-BTBT) films. The resulting device thickness is only ~320 nm, so the device has the ability to adhere well to a three-dimension curved surface. The ultrathin C8-BTBT OTFTs exhibit a mobility as high as 4.36 cm^2^ V^−1^ s^−1^ and an on/off current ratio of over 10^6^. These results indicate the substantial promise of our ultrathin flexible C8-BTBT OTFTs for next-generation flexible and conformal electronic devices.

## 1. Introduction

With the booming demand of microelectronic products, portable electronics, wearable electronics, and even epidermal and bio-implanted electronics have been the emerging research fields in recent decades [1,2,3,4]. These applications incorporate different electronic devices, such as transistors, light-emitting diodes, photodetectors, radio frequency inductors, capacitors, oscillators, and power supplies like the solar cells and wireless coils. Among these various components, organic thin-film transistors (OTFTs) have attracted extensive attention due to their promising application potential in next-generation electronics that include flexible displays [5,6,7,8], radio-frequency identification (RFID) tags [9], electronic skin [10], sensors [11,12], etc. Compared with the inorganic counterparts, their unique advantages such as mechanical flexibility, light weight, and low cost render OTFTs a key component of next-generation electronics [13]. In particular, their compatibility with solution deposition techniques, for example, drop-casting, spin-coating, and printing, reveal OTFTs’ strong potential for large-scale industrial production. The available near-room-temperature solution processes enable semiconductor technology to be realized on plastic substrates, which is promising for flexible and conformal electronics. At the same time, a good adhesion to curved surfaces is expected for OTFTs in future applications. However, this is not easy to achieve for devices with a relative thick substrate, which increases the risk of wrinkles. Reducing the thickness could improve this problem efficiently. Recently, a few outstanding works have been made in fabricating ultrathin and flexible OTFTs. Liu’s groups fabricated 320-nm OTFTs without substrates via a water-floatation delamination [14]. Someya’s group achieved 274-nm OTFTs, resulting from an ultrathin dielectric layer with a thickness of about 64 nm by the chemical vapor deposition (CVD) [15]. Nevertheless, only a few works have deposited the semiconductor layer via solution-processed techniques [16,17]. 

In the past few decades, various solution-based techniques have been reported to achieve high device performances that use different organic materials ranging from small molecules to polymers [18,19,20]. Among these materials, 2,7-dioctyl[1]benzothieno[3,2-b]benzothiophene (C8-BTBT) has attracted considerable interest owing to its good solubility and impressive hole mobility, the highest of which reaches up to 48 cm^2^ V^−1^ s^−1^ [21,22,23]. The alkyl chains introduced to the BTBT core structure can effectively enhance the solubility and ‘‘fasten’’ the adjacent molecules through mutual attractive interactions. However, up to now, almost all of the reported solution-processed C8-BTBT organic field-effect transistors (OFETs) are still deposited on rigid substrates such as Si/SiO_2_ wafers. Currently, only Tsukagoshi’s group has fabricated flexible solution-processed C8-BTBT OFETs on PEN substrates with an average mobility of 0.53 cm^2^ V^−1^ s^−1^ [24]. In contrast, it is desirable to obtain ultrathin C8-BTBT OFETs, which can not only effectively improve the flexibility of the device, but also make the device capable of adhering to a curved surface, presenting the potential for next-generation wearable and implantable electronics. 

In this work, we present the realization of substrate-free flexible C8-BTBT OTFTs on poly(vinyl alchol) (PVA) dielectric via a simple spin-coating method. After the fabrication, the devices were peeled off with the help of 3M tape that was adhered around the device. Compared with the solution-assisted delamination, the dry delamination eliminated the pollution of the solution in the semiconductor material. The resulting device thickness was only ~320 nm. The ultrathin C8-BTBT OTFTs exhibited a mobility as high as 4.36 cm^2^ V^−1^ s^−1^ and an on/off current ratio of over 10^6^, with high reproducibility and good long-term stability. The ultrathin thickness of our C8-BTBT OTFT renders it able to be adhered to a three-dimension curved surface, demonstrating its potential applications in conformal electronics. It is also worth mentioning that there is no extra surface modification to the dielectric layer or post-annealing treatment on the semiconductor layer in our experiment.

## 2. Experimental Section

Device fabrication: Si substrates were cleaned by sonication in acetone and deionized water a few times. Then the Si substrates were immersed in the chromic acid lotion for 10 min, followed by cleaning with flowing deionized water and drying in an ordinary nitrogen atmosphere. The octadecyltrichlorosilane (OTS) treatment proceeded by dipping the Si wafers into OTS solution (OTS:heptane = 1:1000 by volume) for 1 h, after which gate electrodes were deposited on the OTS-modified Si substrate through a shadow mask (20 nm, 0.1 Å/s). PVA (Sigma-Aldrich, Darmstadt, Germany, average Mw ~ 205,000 g/mol) was dissolved in deionized water with different concentrations of 3 wt %, 6 wt %, 8 wt %, and 10 wt %. Then, the solution was stirred at ambient temperature overnight. The PVA solution was spin-coated onto the substrates (5000 rpm, 60 s) and the film was annealed at 70 °C for 3 h in a 0.1 Pa vacuum oven. Subsequently, C8-BTBT (Sigma-Aldrich) dissolved in chloroform (0.2 wt %) was spin-coated (on the center) onto the substrate (5000 rpm, 40 s). Source and drain electrodes were deposited by evaporating (20 nm, 0.1 Å/s) through a shadow mask. Finally, the devices were directly peeled off from the substrates using tape.

Field-Effect Transistor (FET) Characterization: FETs were measured in ambient conditions using a Keithley 4200 SCS semiconductor parameter analyzer. The channel length (*L*) and width (*W*) were 110 μm and 3150 μm, respectively, measured by an Olympus BX51 (Olympus, Tokyo, Japan). Scanning electron microscope (SEM) images were obtained by a Philip XL30 instrument (MicroFEI Philips XL-30 ESEM FEG, Eindhoven, The Netherlands). Atomic force microscopy (AFM) measurements were carried out on a Bruker Dimension Icon instrument (Bruker, Berlin, Germany). Three-dimensional (3D) optical microscope images were measured by Keyence VHX-5000 (Keyence, Japan). The field-effect mobility (*μ*) and threshold voltage (*V*_T_) were extracted from transfer characteristics in the saturated regime by fitting $I_{\mathrm{DS}}=\;C_{i}\mu W/2L{\left(V_{\mathrm{GS}}-V_{T}\right)}^{2}$. The capacitance measurement with frequency was performed using the Au/PVA/Si structure. The measured *C*_i_ (capacitance per unit area) values of the PVA film spun from solutions of different concentrations (3 wt %, 6 wt %, 8 wt %, and 10 wt %) were about 20, 10, 5, and 2 nFcm^−2^ (Figure S1, Supplementary Materials), measured by Keithley 4200 SCS-CUV (Keithley, Beaverton, OR, USA). The average thicknesses of the PVA film were ~139 nm, 283 nm, 606 nm, and 1460 nm, respectively, measured by the XP-2 Profilometer (Ambios Technology, Santa Cruz, CA, USA).

## 3. Results and Discussion

**Figure 1:**
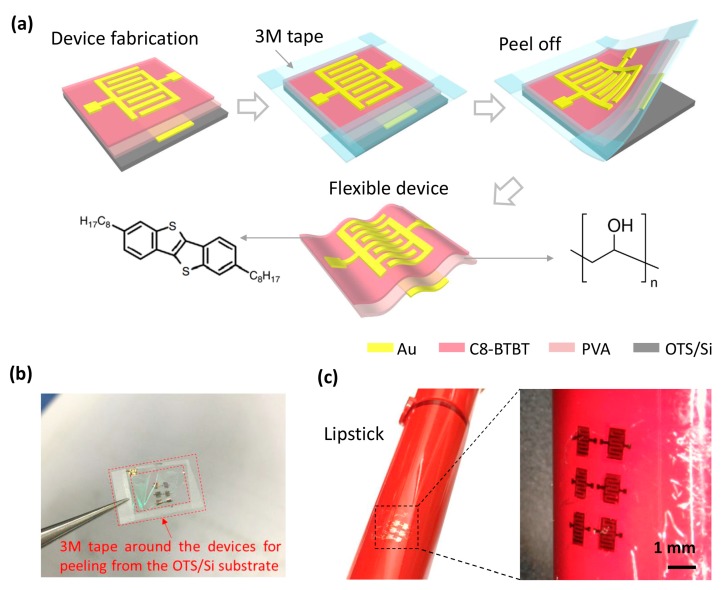
(**a**) Fabrication schematic of an ultrathin C8-BTBT organic thin-film transistor (OTFT) with a bottom-gate top-contact device configuration, and the molecular structures of C8-BTBT and Poly(vinyl alcohol) (PVA). (**b**) Digital photograph of the device. (**c**) Digital photograph of the device adhered on a lipstick tube and 3D optical microscope image of the detail view.

Figure 1a illustrates the fabrication scheme of the ultrathin flexible C8-BTBT OTFT with a bottom-gate top-contact configuration, and the molecular structures of C8-BTBT and PVA. First, the Au gate electrodes were deposited on an OTS-modified Si wafer. Second, a 280-nm poly(vinyl alcohol) (PVA) layer was spin-coated on the substrate as the dielectric layer, and the 20-nm Au gate electrodes were embedded in it. PVA was selected as the dielectric layer due to its promising advantages including low cost, non-toxicity, smooth surface, flexibility, and relatively high dielectric constant [25,26,27]. Most importantly, its good resistance to most organic solvents efficiently avoids the dissolution and swelling of the dielectric layers in the subsequent solution process. Third, C8-BTBT was dissolved in chloroform and spin-coated on the PVA dielectric as the semiconductor active layer. Finally, the device was peeled off directly from OTS/Si substrate with the help of 3M tape that was adhered around the device. Figure 1b shows the real ultrathin devices. The OTS treatment was crucial in the dry delamination process, which provided a hydrophobic surface and hence a weak adhesion between the OTFTs and the substrate. Compared with the solution-assisted delamination, the dry delamination eliminated the pollution of the solution in the semiconductor material. The cross-sectional SEM image (Figure S2, Supplementary Materials) shows that the resulting thickness of the OTFT is ~320 nm, and it is able to conform to a curved surface as shown in Figure 1c. As an example, the C8-BTBT OTFTs were well adhered onto a lipstick tube. 

**Figure 2:**
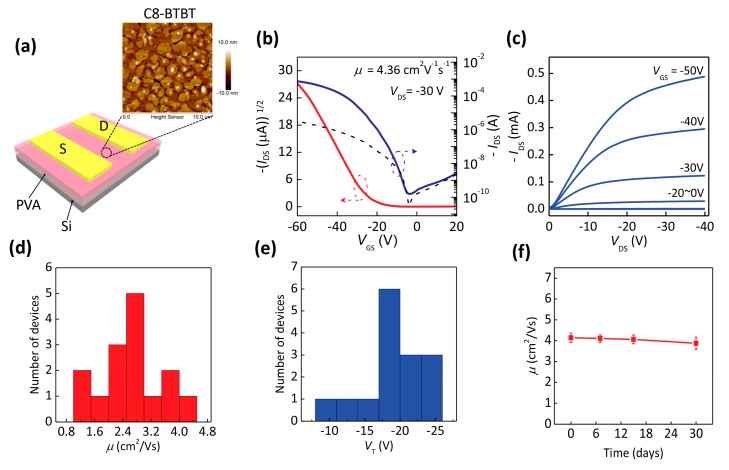
(**a**) Schematic view of the fabricated OTFTs and atomic force microscopy (AFM) image of the C8-BTBT thin film on a PVA dielectric layer. (**b**,**c**) Typical transfer and output characteristics of the device. The dotted line indicates the gate leakage current. (**d**,**e**) Histogram distributions of the mobility (*μ*) and threshold voltage (*V*_T_). (**f**) Dependence relationship of the field-effect mobility on the storage time.

Figure 2a is the schematic image of the C8-BTBT OTFT on the PVA dielectric layer. The inset is the AFM image of the deposited C8-BTBT thin film by spin-coating. The thickness of the film is about 20 nm and the root-mean-square (RMS) roughness value is 2.54 nm. The AFM image shows that the continuous C8-BTBT thin film and the large grain size can be formed on the PVA surface. Figure 2b,c show the typical transfer and output curves of a C8-BTBT OTFT. The transfer curve in Figure 2b indicates the p-type transistor characteristic, and the output curve in Figure 2c presents the typical transistor characteristics with the obvious linear and saturation current regimes. Hysteresis can be observed in our devices (Figure S3, Supplementary Materials), which is a general phenomenon on PVA dielectric layers due to a large mass of hydroxy groups, and could be weakened by modifying the self-assembled monolayers (SAMs) or by adding a cross-linking agent [26,28,29]. By testing 15 devices, the distributions of the mobility and threshold voltage are shown in Figure 2d,e. The device exhibited the highest mobility at 4.36 cm^2^ V^−1^ s^−1^. The average mobility value was as high as 2.64 cm^2^ V^−1^ s^−1^ with a standard deviation of 0.86 cm^2^ V^−1^ s^−1^, and the average threshold voltage was −19.8 V with a standard deviation of 4.02 V. We attribute this relatively high threshold voltage to the large mass of hydroxy groups in the PVA dielectric layer. The applied gate voltage partially aligns the orientations of hydroxyl groups to the direction of the gate electric field, and they would assist the trapping of charges in the OFETs [30]. Hence, a higher voltage would be required to turn on the device. The on/off current ratio of the device was over 10^6^. The field-effect performance of our obtained ultrathin flexible C8-BTBT OTFT on a PVA dielectric layer is obviously higher than the previously reported results for flexible substrates [20,31]. We further tested the devices stored in ambient air (about 25 °C, 20% relative humidity) in Figure 2f. Even after 30 days in ambient air conditions, the changes of mobility amounted to 7%, which verified the relatively long-term stability of the device. 

**Figure 3:**
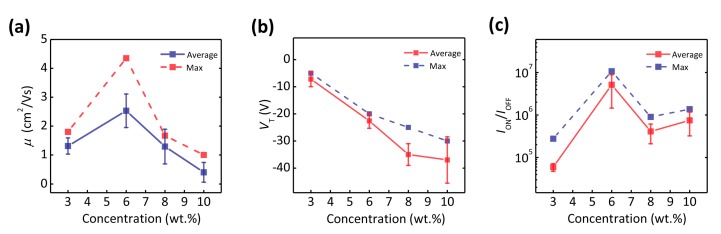
(**a**–**c**) Dependence relationship of the field-effect characteristic parameters: mobility (*μ*), threshold voltage (*V*_T_), and on/off current ratio (*I*_ON_/*I*_OFF_), on the concentration of the PVA solution.

Furthermore, in our experiments we found that the concentration of the PVA solution affected the device performance dramatically. Figure 3 presents the dependence of the field-effect parameters on the different concentrations of the PVA solution. According to the results depicted in Figure 3a–c, the optimized concentration was found to be at 6 wt %. One the one hand, the effect of the concentration of the PVA solution on the field-effect performance of the devices is related to the thickness of the PVA dielectric layer. At a low concentration (3 wt %), due to the ultrathin thickness of the PVA layer (~140 nm), the PVA dielectric layer has a poor dielectric breakdown strength, and a large leakage current and off-current are induced (Figure S4, Supplementary Materials) which results in the low success ratio of device fabrication as well as the low mobility and on/off current ratio, as shown in Figure 3a,c. With the concentration increasing, the thickness of the spin-coated PVA films increases dramatically, resulting in a decreased capacitance (Figure S1, Supplementary Materials). For the thicker PVA dielectric layer, the induced charge density in the conductive channel is decreased at the same gate voltage, hence a higher voltage is required to turn on the device. At the same time, less induced charges can be contributed to the carrier transport, resulting in a lower field-effect mobility. 

**Figure 4:**
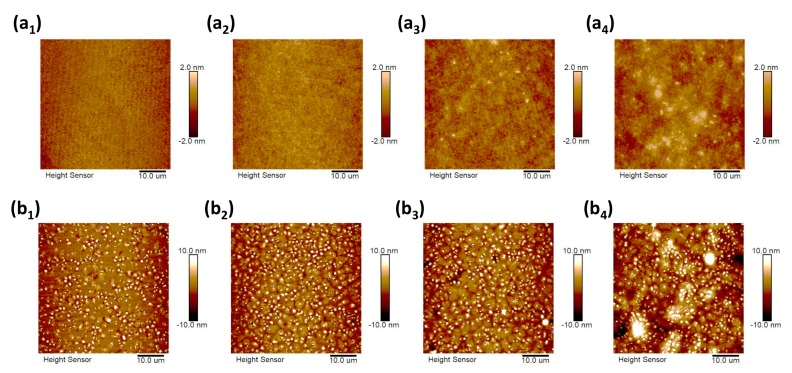
(**a_1_**–**a_4_**) AFM images of the PVA films spun from different concentrations (3 wt %, 6 wt %, 8 wt %, and 10 wt %). (**b_1_**–**b_4_**) AFM images of the C8-BTBT films on the corresponding PVA films in (**a_1_**–**a_4_**).

On the other hand, the effect of the concentration of the PVA solution on the field-effect performance of the devices is related to the morphology of the PVA dielectric layer. It is well known that the morphology of the film affects the device performance greatly. We further investigated the AFM images of the PVA and C8-BTBT films. Figure 4(a_1_–a_4_) correspond to the PVA films spin-coated with different concentrations (3 wt %, 6 wt %, 8 wt %, and 10 wt %). The RMS roughness values of the PVA films were 0.396, 0.430, 0.465, and 0.681 nm, respectively. This indicates that the surfaces of the PVA films spin-coated with the low concentrations of 3 wt % and 6 wt % are relatively smooth. When the concentration increases, the surface morphology presents an obvious inhomogeneity which can further influence the morphology of the semiconductor layers deposited on them. The resulting morphology of the C8-BTBT films is shown in Figure 4(b_1_–b_4_), and the RMS roughness values were found to be 3.43, 3.53, 3.99, and 5.17 nm, respectively. With the increased concentration, more crystalline aggregates and a slightly lager grain size was obtained (magnified AFM images, see Figure S5, Supplementary Materials). In particular, at the concentration of 10 wt %, the large-size crystalline aggregates could be obviously observed. These aggregates between the domains could disturb the charge transport from one domain to another [32]. We speculate that the morphology is also one of the main factors influencing the device performance. The thin, smooth C8-BTBT films with less crystalline aggregates was favorable for the improved carrier transport, which combined the thickness effect as mentioned above, resulting in an optimized concentration of PVA solution at 6 wt %. 

**Figure 5:**
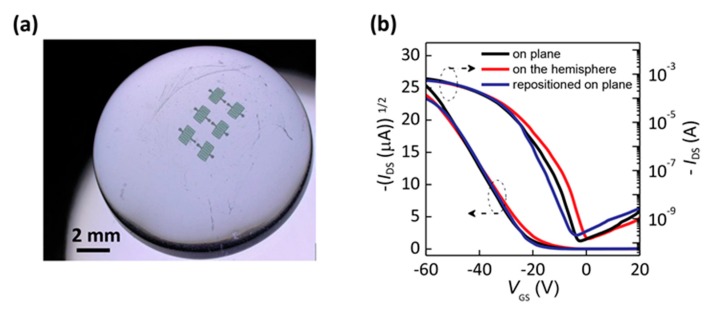
(**a**) 3D optical microscope image of ultrathin C8-BTBT OTFTs adhered on a glass hemisphere (*r* = 6.5 mm). (**b**) Corresponding transfer characteristics of the device under three deformation states: on plane, on the glass hemisphere, repositioned on plane.

In order to further show the adherence capability of our ultrathin flexible OTFTs on the three-dimension curved surface, we tested the device under three different deformation states. We first put the device on a flat surface and then transferred it onto a glass hemisphere with a radius of 6.5 mm, and finally put it on a flat surface again. Figure 5a shows the 3D optical microscope image of the devices well adhered to the glass hemisphere. The corresponding electrical characteristics of the device are shown in Figure 5b. The calculated field-effect parameters are presented in Table 1. The mobility decreased slightly from 3.43 cm^2^ V^−1^ s^−1^ to 3.04 cm^2^ V^−1^ s^−1^, and the threshold voltage decreased when the device was transferred from the flat surface to the 3D curved surface. The subthreshold swing (SS) value, which is defined as the *V*_GS_ change needed to induce an *I*_DS_ change of one order of magnitudes and indicates the speed of the transition between off and on states, was a little higher than the previously reported results [22,33]. This could be induced by the polar hydroxy groups in the PVA layer. When the device was repositioned on a flat surface, the field-effect performance showed a negligible change. This indicated that no obvious damage occurred to our ultrathin device during the peeling process and adhesion, which benefitted from the ultrathin thickness. These results further indicate that high-performance ultrathin OTFTs show strong potential for next-generation flexible and conformal electronic devices.

## 4. Conclusions

In summary, we fabricated ultrathin flexible OTFTs based on spin-coated C8-BTBT films via a simple dry peeling technique. The thickness of the device was ~320 nm. The ultrathin OTFTs exhibited a mobility as high as 4.36 cm^2^ V^−1^ s^−1^ and an on/off current ratio of over 10^6^. The ultrathin flexible device could adhere well to a three-dimension curved surface. When the device conformed to a glass hemisphere with a radius of 6.5 mm, the device performance showed negligible change, which benefitted from the ultrathin thickness. These results effectively indicate that our ultrathin flexible OTFTs show promising potential for applications in flexible and conformal electronic devices. 

## Figures and Tables

**Table 1:** The calculated field-effect parameters of the C8-BTBT OTFT under three deformation states.

Deformation	*μ* (cm^2^/Vs)	*V*_T_ (V)	*SS* (V/dec)	*I*_ON_/*I*_OFF_
on plane	3.33	−16	4.25	6.85 × 10^6^
on the hemisphere	3.04	−13	4.34	4.34 × 10^6^
repositioned on plane	3.23	−14	4.07	4.06 × 10^6^

## Supplementary Materials

The following are available online at www.mdpi.com/1996-1944/10/8/918/s1, Figure S1: Dependence of the capacitance with frequency of the PVA dielectric layer spun from solution of different concentrations (3 wt %, 6 wt %, 8 wt %, and 10 wt %), Figure S2: The cross-sectional SEM image of the device. The red line indicates the “S/D electrode (20 nm) + C8-BTBT (~20 nm)” layer and the yellow line indicates the PVA layer (~280 nm) with embedded gate electrodes, Figure S3: (a) Transfer curves swept in backward and forward directions of C8-BTBT OTFTs on PVA dielectric layer, Figure S4: (a–d) The transfer characteristics of C8-BTBT OTFTs on PVA dielectric layer spun from solution of different concentrations (3 wt %, 6 wt %, 8 wt %, and 10 wt %), Figure S5: (a1–a4) AFM images of C8-BTBT films on corresponding PVA films spun from different concentrations (3 wt %, 6 wt %, 8 wt %, and 10 wt %).
