# Supplementary material for: 320-nm Flexible Solution-Processed 2,7-dioctyl[1] benzothieno[3,2-b]benzothiophene Transistors
Source: Materials (Basel). 2017 Aug 9;10(8):918. doi: 10.3390/ma10080918 (PMC5578284; doi:10.3390/ma10080918)
Supplement: Supplementary file 1 [file materials-10-00918-s001.pdf]

Supplementary Materials: 320-nm Flexible Solution-Processed 2,7-dioctyl[1] benzothieno[3,2-b]benzothiophene Transistors

Hang Ren, Qingxin Tang, Yanhong Tong and Yichun Liu

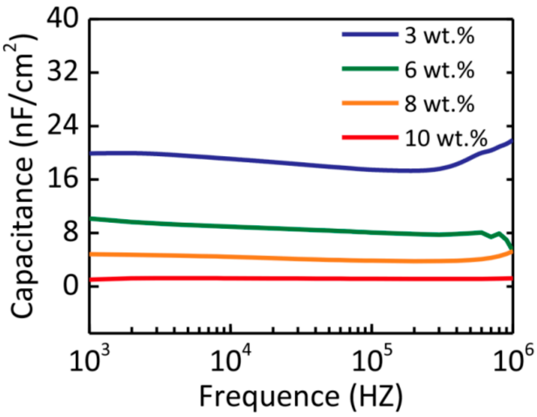

Figure S1. Dependence of the capacitance with frequency of the PVA dielectric layer spun from solution of different concentrations (3 wt %, 6 wt %, 8 wt % and 10 wt %).

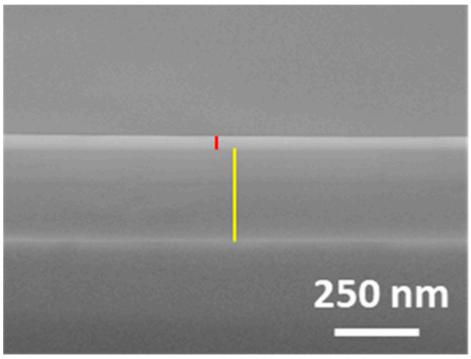

Figure S2. The cross-sectional SEM image of the device. The red line indicates "S/D electrode (20 nm) + C8-BTBT (~20 nm)" layer and the yellow line indicates the PVA layer (~ 280 nm) with gate electrode embed in it.

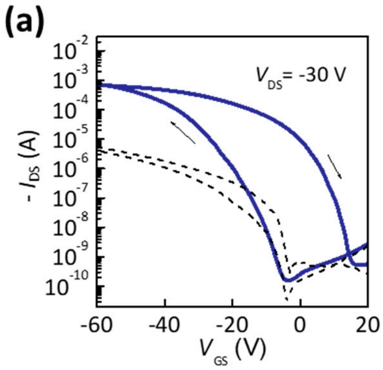

Figure S3. (**a**) Transfer curves swept in backward and forward directions of the C8-BTBT OTFT with PVA dielectric layer.

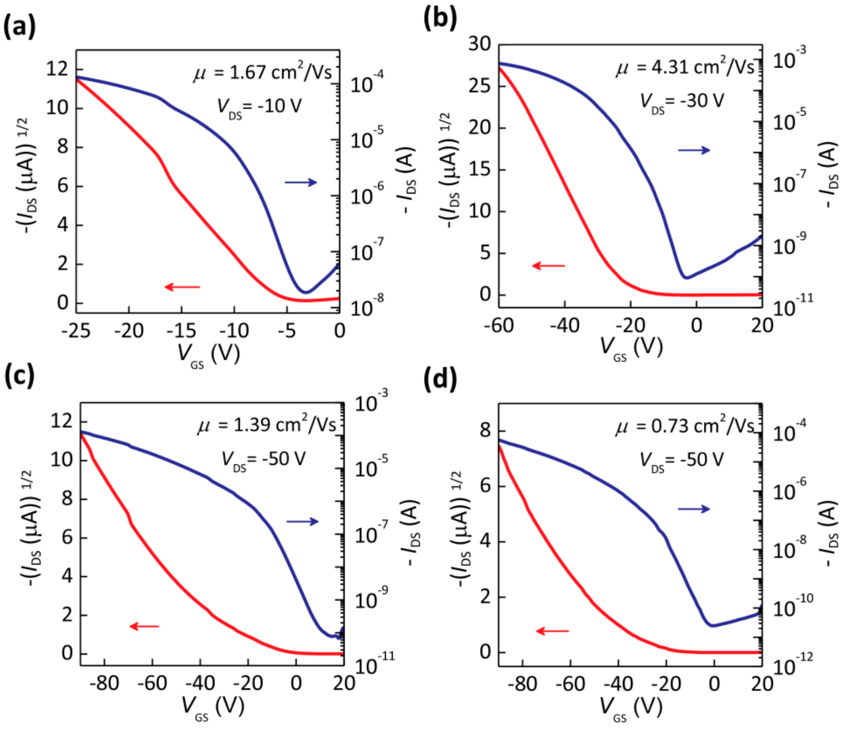

Figure S4. (**a**–**d**) The transfer characteristics of C8-BTBT OTFTs with PVA dielectric layer spun from solution of different concentrations (3 wt %, 6 wt %, 8 wt % and 10 wt %).

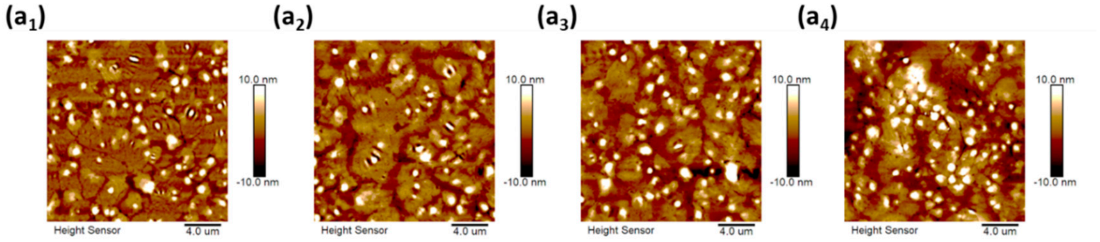

Figure S5. (**a1**-**a4**) AFM images of the C8-BTBT films on the corresponding PVA films spun from different concentrations (3 wt %, 6 wt %, 8 wt % and 10 wt %).
